# Cross Layer Design for Optimizing Transmission Reliability, Energy Efficiency, and Lifetime in Body Sensor Networks

**DOI:** 10.3390/s17040900

**Published:** 2017-04-19

**Authors:** Xi Chen, Yixuan Xu, Anfeng Liu

**Affiliations:** School of Information Science and Engineering, Central South University, Changsha 410083, China; xchen@csu.edu.cn (X.C.); yixuan_xu@csu.edu.cn (Y.X.)

**Keywords:** wireless body area networks, cross layer optimal, low delay, energy efficiency, lifetime

## Abstract

High transmission reliability, energy efficiency, and long lifetime are pivotal issues for wireless body area networks (WBANs). However, these performance metrics are not independent of each other, making it hard to obtain overall improvements through optimizing one single aspect. Therefore, a Cross Layer Design Optimal (CLDO) scheme is proposed to simultaneously optimize transmission reliability, energy efficiency, and lifetime of WBANs from several layers. Firstly, due to the fact that the transmission power of nodes directly influences the reliability of links, the optimized transmission power of different nodes is deduced, which is able to maximize energy efficiency in theory under the premise that requirements on delay and jitter are fulfilled. Secondly, a relay decision algorithm is proposed to choose optimized relay nodes. Using this algorithm, nodes will choose relay nodes that ensure a balance of network energy consumption, provided that all nodes transmit with optimized transmission power and the same packet size. Thirdly, the energy consumption of nodes is still unbalanced even with optimized transmission power because of their different locations in the topology of the network. In addition, packet size also has an impact on final performance metrics. Therefore, a synthesized cross layer method for optimization is proposed. With this method, the transmission power of nodes with more residual energy will be enhanced while suitable packet size is determined for different links in the network, leading to further improvements in the WBAN system. Both our comprehensive theoretical analysis and experimental results indicate that the performance of our proposed scheme is better than reported in previous studies. Relative to the relay selection and power control game (RSPCG) scheme, the CLDO scheme can enhance transmission reliability by more than 44.6% and prolong the lifetime by as much as 33.2%.

## 1. Introduction

Developments in sensors have caused wireless portable devices to spring up nowadays, making human-centered wireless sensor network a hot research topic. Body Sensor Networks (BSNs) are collections of wearable (programmable) sensor nodes communicating with a local personal device (or coordinator), and have emerged as a revolutionary technology in many application domains in health-care, fitness, smart cities, and many other compelling Internet of Things (IoT) applications [1,2,3,4]. The so-called wireless body area networks (WBANs) also belong in this environment [5,6,7]. Due to their characteristics of practicability and portability [8,9,10], WBANs have promising applications in many fields, such as health care, amusement, and the military [3,11,12,13,14,15]. The concept of WBANs was first raised by Zimmerman in 1996 [16], and since then many studies have been published, focusing on using low-powered wireless sensor network to collect vital sign data of humans [17,18,19,20,21]. These studies greatly facilitate the monitoring of personal health in different environments and improve the quality of human life.

Although WBANs stem from wireless sensor networks (WSNs) [22,23,24], due to their special applications, they differ from WSNs in many aspects [25,26,27,28,29]. Firstly, high energy efficiency is an urgent need for WBANs [29,30]. Specifically, in WBANs, sensors are powered by batteries and have very limited energy [1,3,29,30]. Furthermore, WBANs have stricter requirements on the size of sensors since they are placed on the human body, which also leads to a decrease in battery size [31]. Therefore, compared with other kinds of wireless sensor networks, the energy of sensors in WBANs is highly limited, making it more important to design a system with high energy efficiency [29,30,32]. As with WSNs, a great proportion of the energy consumption in WBANs is used for communication [33,34,35]. Therefore, one way to increase the energy efficiency and prolong the lifetime of a network is by maintaining efficient communication between nodes [33,34,35]. For example, sensor nodes can transmit data to a hub in single-hop or multi-hop style [36,37,38]. Single-hop style specializes in topology briefness. However, it cannot guarantee reliability in transmission over a long distance. Compared with single-hop, the multi-hop style leads to a more complex WBAN topology and more overall data size but without weakness in long-range transmission reliability.

Apart from high energy efficiency [1,3,11,12,29,30,32], WBANs also have special requirements on the performance of the network. Take applications related to human life as an example (e.g., remote surgery, life-critical or medical disaster applications), such applications tend to have a requirement of low delay because of potential irreversible losses caused by high delays [39]. For example, WBANs should respond to emergencies as soon as possible when used to monitor people with heart diseases. Therefore, apart from high energy efficiency, performance metrics such as lifetime, delay, and reliability (referred to as QoS requirements) are also critically important [40].

Although there has already been studies aimed at optimizing the energy efficiency and QoS requirements for WBANs [29,30,40], several key issues deserve further study: (1) Most research has focused on optimizing the performance from one single aspect, leading to limited space for improvements (e.g., adjustments of duty cycle, size of contention window, and algorithms for choosing relay nodes). Intuitively, better performance can be achieved through optimizing several aspects simultaneously. However, the interrelations between them make this more complex and harder in practice; (2) Although much research on improving energy efficiency exists, most of it ignores the impact of packet size on energy efficiency [4]. Besides, packet size also influences the reliability and delay during transmission. Therefore, the problem of choosing a suitable packet size to improve the performance also deserves further study; (3) The final target of optimizing energy consumption in WBANs is to prolong the lifetime of a network instead of reducing the energy consumption of nodes. From the view of multi-hop WBANs, nodes that are near the hub consume much more energy than remote nodes since apart from transmitting back their own data, they also need to forward data generated by remote nodes. In other words, remote nodes tend to have more residual energy. Generally speaking, with enough energy, performance on QoS requirements can be effectively improved through methods like enhancing transmitting power.

In this paper, we conduct a comprehensive study on problems ignored by previous studies. Its main contributions include:

1. We obtain the transmission power of different nodes that maximizes their energy efficiency through theoretical analysis.
2. A Cross Layer Design Optimal (CLDO) scheme is proposed to simultaneously improve the transmission reliability, energy efficiency, and lifetime of WBANs through optimizing the network layer, medium access control (MAC) layer, and PHY layer:
  - The CLDO scheme first optimizes the transmission power for every link in the WBAN from the MAC layer and PHY layer, which can maximize the energy efficiency in theory under the premise that requirements on delay and jitter are assured to be met.
  - After that, a relay decision algorithm is proposed to optimize the process of selecting relay nodes from the network layer, which burden the routing task for sensor nodes.
  - Since different locations of nodes lead to unbalanced energy consumption, then CLDO scheme then will enhance the transmission power of nodes with more residual energy to further improve QoS requirement performance. Similar to step 1, this operation is related to the MAC layer and PHY layer.
  - Finally, the CLDO scheme chooses suitable packet sizes for different data packets transmitted in the network to maximize energy efficiency, which involves the network layer, MAC layer, and PHY layer simultaneously since the actual header consists of their sub-headers.
3. Through our extensive simulation, we demonstrate that both the transmission reliability and the lifetime of a WBAN can be enhanced using the proposed CLDO scheme. Compared with previous methods, the transmission reliability reaches more than 44.6% while the lifetime can be prolonged by 33.2%.

The rest of this paper is organized as follows: in Section 2, related works are reviewed. The system model and problem statement are described in Section 3. In Section 4, design details of the CLDO scheme are presented. A theoretical analysis on the performance of the proposed CLDO scheme is given in Section 5. Section 6 gives the experiment results and comparisons. We conclude in Section 7.

## 2. Related Work

This section presents previous work related to optimizing energy efficiency and QoS requirements from different layers of WBANs, including optimization in the MAC layer and network layer, along with interrelations between data packet size and the performance of the network.

### 2.1. Optimizations in the MAC Layer

Due to the limited size of sensors in WBANs, they also have smaller resources, such as battery and storage [1,3,29,30,40]. Therefore, effective utilization of energy is a main challenge for WBANs. One important source of energy consumption is data communication [17]. Specifically, even without the need to transmit data, nodes also need to intercept the channel. On the other hand, when nodes are sleeping, energy consumption is far smaller than that when nodes are active. Therefore, to effectively improve energy efficiency, nodes should be in sleep mode when there is no need for them to transmit and receive data, i.e., adopt a duty cycle mechanism. However, it is possible for one node to be in sleep mode when other nodes choose it as their relay node. In this case, this node will first need to awake before forwarding data, causing the delay of the network to increase. Therefore, a careful design of the size of the duty cycle is necessary. In detail, with a small duty cycle, more energy can be saved. However, a relatively large delay also arises in the network.

Wang et al. analyzed specific situations in WBANs and proposed an alternative method for selecting the most appropriate sensors to activate based on some knowledge with respect to diseases and their corresponding vital signs [15,41]. Due to the fact that redundancy exists among data transmitted by different nodes, there is no need for all nodes to be active provided that global information can be deduced from local information [13]. With this method, only nodes with the ability to obtain global information are active while others are in sleep mode. According to their theoretical analysis and experiments, energy can be saved under the premise that monitored information is not influenced.

Shu et al. proposed a hybrid medium access protocol with an interrupt mechanism (I-MAC) to enhance the energy and time slot utilization efficiency and to satisfy the data delivery delay requirement simultaneously [42]. In their protocol, time slots can be divided into two different categories. The first kind is normal time slots mainly used to deliver periodic data while another kind uses a superframe structure with a longer length, aiming at delivering real-time data. Due to a longer length, transmission of real-time data can be finished within one slot.

To maximize the network lifetime of WBANs, one common and effective solution is to deploy extra relay nodes in the network. Cai et al. proposed an adaptive MAC protocol named Network Longevity Enhancement by Energy Aware medium access control Protocol (NLEEAP) [43], which has the capacity to decrease energy consumption without introducing additional devices. In the most common case, NLEEAP will adopt single-hop transmission. However, when nodes have limited residual energy, those relay nodes will activate and transmit data with multi-hop routing [14]. NLEEAP also adopts one kind of superframe to ensure that the network is still able to function properly when switching to multi-hop transmission.

### 2.2. Optimizations in the Network Layer

Optimizations in the network layer mainly focus on schemes related to routing, which have the responsibility of transmitting data from source nodes to the hub in single-hop or multi-hop style. Many challenges exist during transmission, for example, transmitted data may suffer from security attacks. Besides, QoS requirements also need to be taken into consideration [40,44,45,46]. Therefore, routing schemes have attracted wide interest in wireless sensor network studies [8,9]. Original schemes for routing take distances from the hub into consideration, where nodes tend to choose the neighbor node with minimum distance from the hub as their relay nodes [10,24]. However, later studies discovered that these schemes will cause energy consumption of important nodes in the network to be much higher than other nodes. Therefore, schemes proposed after this discovery not only take distances into consideration, but also the energy consumption of nodes. Specifically, a weight factor that is able to reflect distances and energy consumption simultaneously is adopted to facilitate the selection of relay nodes [40]. Ayatollahitafti et al. proposed a protocol that utilizes hop counts and link cost of neighboring nodes to select the best next hop for packets forwarding [40]. In their protocol, link cost of neighboring nodes synthesizes following factors: available queue size, link reliability, and residual energy. Each node will select an appropriate node among neighboring nodes as the next hop node, which minimizes hop counts to the sink and maximizes link cost [47]. According to [40], routing schemes in BANs can be categorized into five groups, namely: delay tolerant, QoS aware, cross layer, thermal-aware, and cluster based routing. Besides, collaborative communication also improves the performance of WBANs.

### 2.3. Relationships between Packet Size and Network Performance

Selection of optimal packet size is considered in [48] for the enhancement of energy efficiency in BANs as there tend to be complex relationships between packet size and performance on energy efficiency, reliability, and delay: (1) The ratio of the effective length of data packet to the total length of data packet is known as payload ratio. Due to the fixed length of packet headers, the payload ratio of data packets is expected to improve with increasing packet size. Besides, the proportion of energy used to transmit effective data also increases, leading to an improvement on energy efficiency; (2) Apart from energy efficiency, the packet size also influences the reliability of transmission [49]. With fixed transmitting power, the error rate of data packets will increase with a growth on packet size, leading to a decrease of the transmission reliability. On the other hand, the success rate of transmission is expected to increase with a smaller packet size. However, since more data packets need to be transmitted with a smaller packet size, the overall success rate can still be lower than that of transmitting large size data packets; (3) The overall success rate will directly influence the transmission delay. Therefore, packet size also has an impact on the transmission delay.

## 3. System Model and Problem Statement

In this section, an overview of our system model is provided, and then the problems to be solved are presented.

### 3.1. System Model

In our framework, WBAN consists of n sensor nodes deployed on a human body, which are used to transmit sensed healthcare data to the hub of the WBAN. Then, the hub can further forward the collected data to a server outside the body. Each sensor node is regarded as a GI/G/1 queue to handle affairs, i.e., one node deals with one packet at a time on a first-come, first-served basis [30]. To guarantee the effectiveness and efficiency of transmission in the WBAN, sensor nodes are deemed to have the capacity on either directly connect to the hub or connect to it through a multi-hop path. Under the second situation, middle nodes act as relays to forward collected data. The connection between each node i and its relay j can be denoted as a link(i,j). Moreover, we assume that the hub has fast processing speed and sufficient energy resources to perform the necessary computation for data flow planning. It can execute power level assignments, packet size assignments and relay assignments for all sensor nodes in WBAN simultaneously [29].

Sensor nodes in a WBAN are equipped with the same initial battery energy $e_{battery}$. Yet, considering the vital role of hub in WBAN, relatively unexhausted energy is provided for it, which can be realized so long as the hub is a chargeable device, such as a telephone or another personal digital assistant. Owing to nodes’ discrepancy in categories and applications, each of them generates data packets according to an individual speed obeying a Poisson distribution with expected value $v_{i}$, in turn, the inter-arrival times of self-generating packets obeys exponential distribution, with expected value $v_{i}^{-1}$ and variance $v_{i}^{-2}$, taking default packet size N as a unit.

In the data collection process, sensed data are integrated into data packets and transmitted over links [50]. To be in accord with a realistic WBAN situation, packet size is limited to a finite set of discrete values, and so is transmission power. Besides, each packet has a fixed header to facilitate the transmission [29].

As to wireless channel conditions, a commonly used log-normal fading model is built for on-body WBAN channels [21,32]. In the model, signal attenuation caused by geometric signal spread is included. Furthermore, small-scale fading experienced by channels is also considered, which reflects the influence of transmission fluctuations coming from the arrival of multiple simultaneous signals. Hence, the Signal to Noise Ratio (SNR) $\gamma_{u,v}$ from a sensor node u to another node v obeys a log-normal distribution, as described below: number this equation

$$f\left(\gamma_{u,v}\right)=\frac{1}{\sqrt{2\pi }\gamma_{u,v}\sigma_{u,v}}\exp\left(\frac{-{\left(\gamma_{u,v}^{\mathrm{dB}}-\mu_{u,v}\right)}^{2}}{2\sigma_{u,v}^{2}}\right)$$

where $\mu_{u,v}$, $\sigma_{u,v}$, and $\sigma_{u,v}^{2}$ represent the mean, the standard deviation and the variance of the SNR $\gamma_{u,v}$ in dB, respectively. In addition, non-coherent differentially encoded binary phase-shift keying (DBPSK) modulation is employed for reliable transmission in the channel.

**Figure 1:**
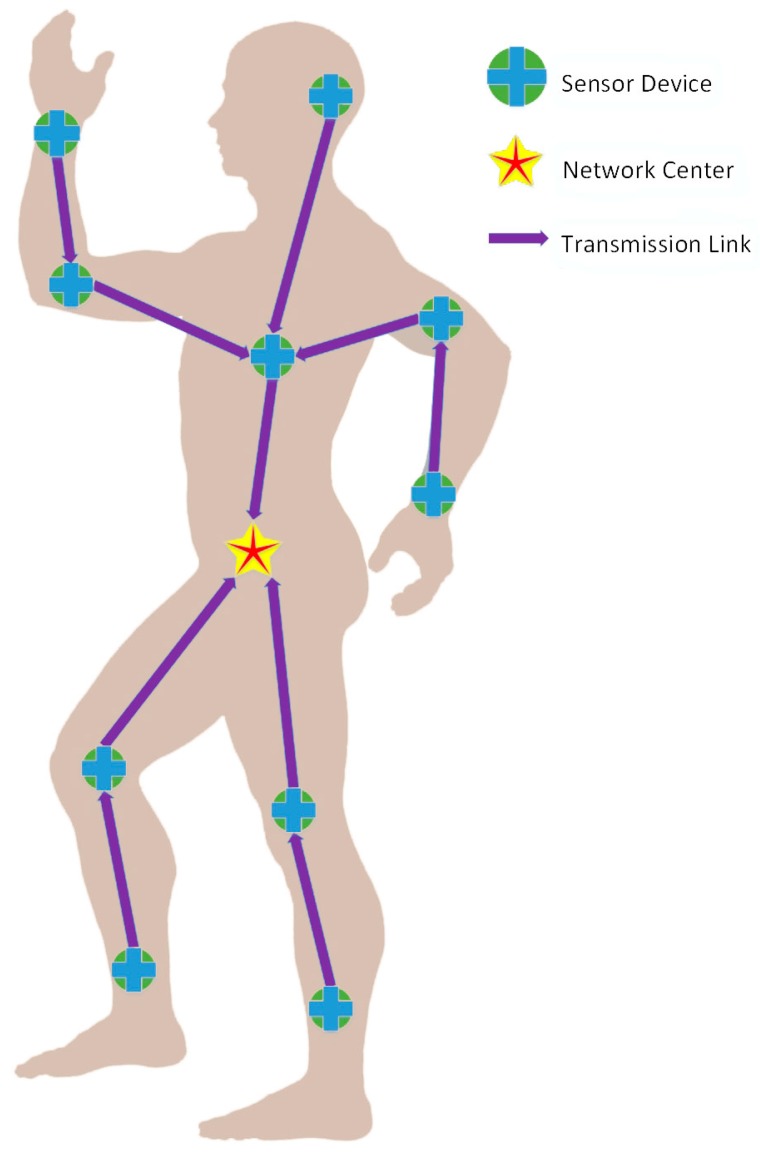
The WBAN architecture.

Furthermore, slotted Aloha access is used for the contended allocations in the WBAN, in other words, each node utilizes slotted Aloha to get access to the shared medium, which has already been exhaustively interpreted in [23]. We use the symbol q to denote contention probability in the Aloha system. Figure 1 later this section illustrates the WBAN architecture.

### 3.2. Problem Statement

The goal of our scheme is to prolong the lifetime of the WBAN, enhance transmission reliability in the process of data transmission, and balance the distribution of energy utilization in the WBAN.

**Definition** **1.** *The lifetime of WBAN (denoted as* $T_{life}$*) is defined as the time intervals from the time when the WBAN is established to the time when the first dead node appears, which results from exhausted battery energy. Hence,* $T_{life}$ *is determined by the energy consumption speed of each node* $C_{i}$ *(*i∈n*).*

Thus, maximizing the lifetime can be translated into minimizing transmission power of the first dead sensor node, or the crest value of energy consumption speed $C_{max}$, as depicted in the following formula:

(1) $$\;\max T_{life}=\max\min\left(e_{battery}/C_{i}\right),\;i\in n$$

**Definition** **2.** *Transmission Reliability (denoted as* $R_{i}$ *for packet transmitted from node* i *to the hub) is defined as the probability of completing packet transmission with success in the path.*

Suppose the nodes in the multi-hop path from node i to the hub constitute one set $Route_{i}=\left\{n_{i,1},\;n_{i,2},\ldots ,\;n_{i,k}\right\}$, where $n_{i,1}$ refers to the first node in the path, i.e., node i itself, and $n_{i,k}$ is the k-th node in the path. Separately, the transmission reliability of node $n_{i,k}$ to forward packet is $R_{i,k}$.

Consequently, the goal of minimizing transmission reliability can be expressed as:

(2) $$\max\begin{array}{c} R_{i} \end{array}=\max\overset{k}{\underset{j=1}{\sum }}R_{i,j}\;s.t\;i\in n$$

**Definition** **3.** *Energy utilization equilibrium rate (denoted as* χ*) refers to the minimum ratio of each node’s utilized energy when the WBAN dies to its initial energy. Our purpose is to maximize the utilization equilibrium rate, so that battery energy in sensors nodes can be fully exploited. Since average energy consumption speed is constant for every individual node if the topology of WBAN and other parameters related to data transmission are decided, the aforementioned ratio is equivalent to the ratio of the minimum energy consumption speed of nodes* $C_{min}$ *to the maximum consumption speed of nodes* $C_{max}$*. Therefore, maximizing the ratio is depicted as:*

(3) $$\max\;\chi =\max\frac{C_{min}}{C_{max}}$$

Obviously, with the optimization of the CLDO scheme, our target is to maximize the lifetime of the WBAN, maximize the transmission reliability of each node, and maximize the energy utilization equilibrium rate, summarized as:

(4) $$\begin{array}{c} \max\;T_{life}=\min\max\;C_{i}\;s.t\;i\in n\;\\ \max\begin{array}{c} R_{i} \end{array}=\max\overset{k}{\underset{j=1}{\sum }}R_{i,j}\;s.t\;i\in n\\ \max\;\chi =\max\frac{C_{min}}{C_{max}}\; \end{array}$$

## 4. Design of the CLDO Scheme

### 4.1. Research Motivation of CLDO Scheme

The research motivations of the CLDO scheme are based on our comprehensive studies related to WBANs, which can be summarized in the following four observations:

#### 4.1.1. Observation 1

**Figure 2:**
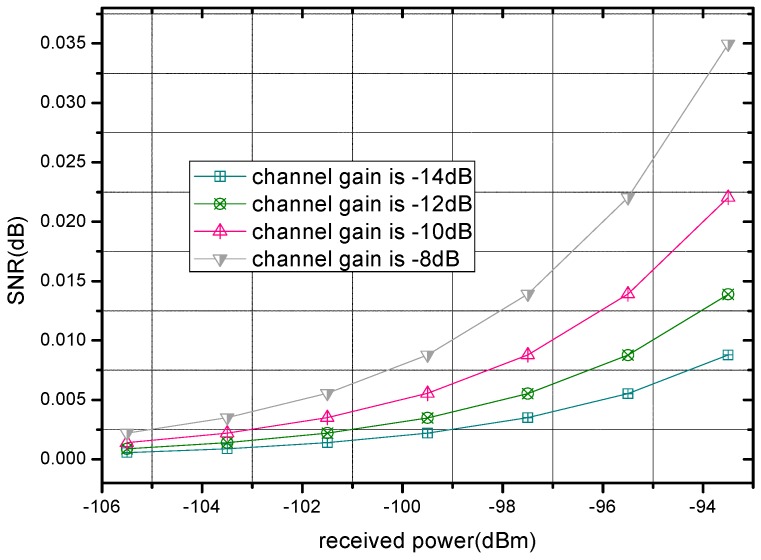
The SNR of channel with different received power.

Transmission power control has a significant influence on the network lifetime transmission reliability. In a WBAN, transmission power of a sensor node is a vital measure of its energy consumption, which has direct impacts on the WBAN’s lifetime. Hence, one effective way to prolong the network lifetime is by reducing the transmission power. However, a decrease in the transmission power leads to increased signal attenuation caused by noise and other disturbance, and eventually this leads to a SNR reduction. As a result, packet error and loss appear more frequently during transmission, i.e., transmission reliability is reduced. The specific relation between power and SNR is given in Equation (19). Moreover, since high real-time and stable performance is usually required in WBANs for special functions with respect to human healthcare and security, low delay and jitter over links are needed. Both of them also increase with more frequent packet error and loss. Based on the aforementioned descriptions, the optimization of power decision for sensor nodes is necessary. Figure 2 illustrates the relation between the received power and SNR with different channel gain, which is one important reflection on channel conditions. Obviously, SNR rises with the augment of transmission power since the channel condition is improved.

**Figure 3:**
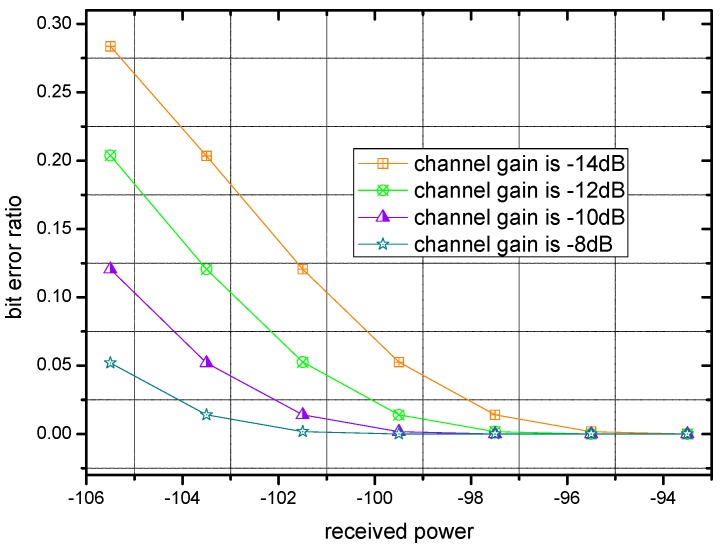
Bit error ratio of transmission with different received powers.

Bit error ratio with different received power are shown in Figure 3, where each curve corresponds to an individual channel gain. It is clear that in Figure 3, the bit error ratio descends with raising power.

#### 4.1.2. Observation 2

In addition to transmission power, packet size is another critical factor that determines the quality of transmission, which can be concretely described as follows: on the one hand, to reach same transmission success rate, large packets require higher power than small packets because of the increased difficulty of ensuring more bits will be transmitted correctly. On the other hand, since each packet has a fixed header to inform the collection process apart from its original size, data size sensed from nodes themselves spontaneously obtain a higher percent in large packet than small packets. As a result, the total data size flowing in WBAN varies with the change of packet size, which affects transmission delay and jitter directly. Therefore, it is necessary to conduct research on the packet size decision for links in WBANs to obtain performance optimization. 

**Figure 4:**
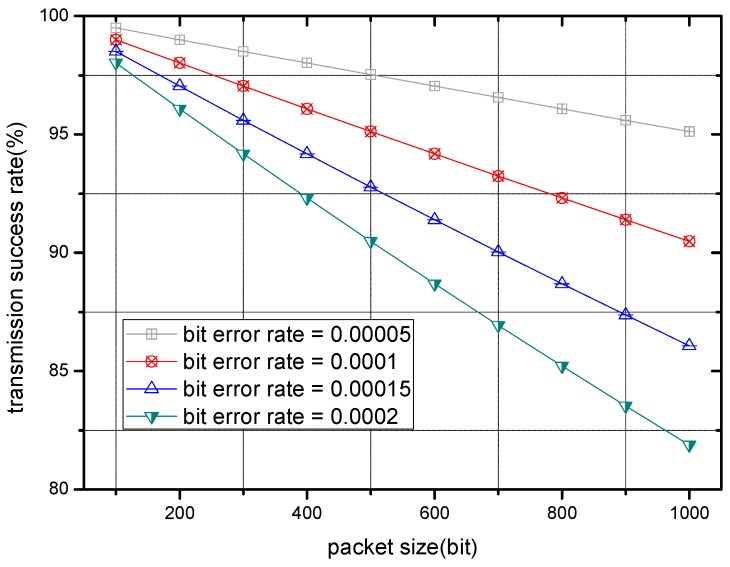
Transmission success rate with different packet size.

Figure 4 illustrates the relation between packet size and transmission success rate. As each curve shows, with rising packet size, the transmission success rate descends conversely, which is consistent to the analysis above.

**Figure 5:**
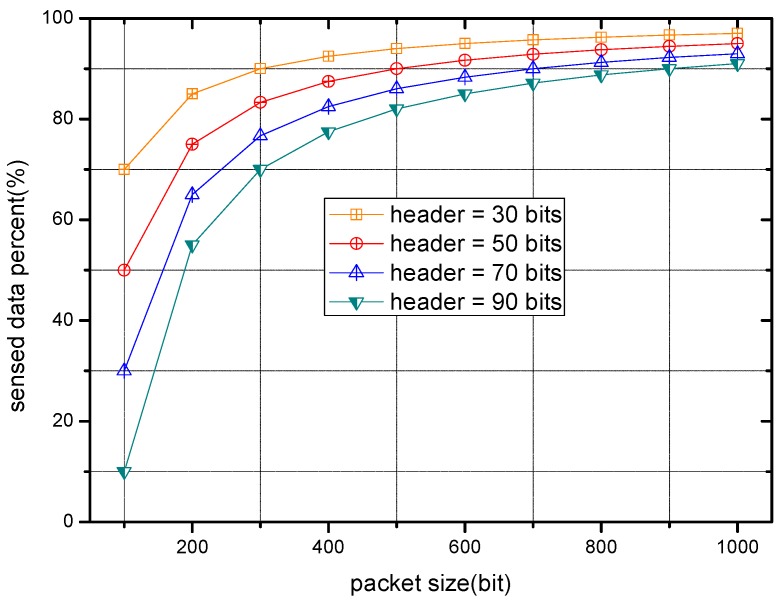
The percent of sensed data with different packet size.

The percentages of sensed data in packets with different packet size are presented in Figure 5. It is clear from Figure 5 that when the packet size is small, e.g., 100 bits, the sensed data occupy a limited percent in the packets. However, this percentage keeps ascending with the growth of packet size. Moreover, when the packet size is large enough, the percentage of sensed data remains relatively stable with different header size, which can be interpreted as a negligible role played by data size of the header in packets at this time.

#### 4.1.3. Observation 3

Since WBANs are deployed on bodies with a specific human structure and physical environment, one sensor node cannot simply select its nearest node as the relay to hub in the multi-hop transmission path. One common and practical method is to select relay nodes with the highest energy efficiency as candidates for transmitting packets [7]. In this way, however, sensor nodes tend to choose several fixed nodes as relays to obtain optimal energy efficiency performance of the WBAN. As a result, even if those chosen nodes perform well on energy efficiency, they converge most of the sensed data after the network completes the topology modification. Under this situation, they are responsible for much more data load than other nodes, which in turn increases the transmission delay, accelerates nodes’ death and inevitably aggravates the unbalanced distribution of energy consumption in the network. Based on the aforementioned description, a further optimization of relay decisions can be obtained to trade off between the energy efficiency and the energy consumption distribution equilibrium.

**Figure 6:**
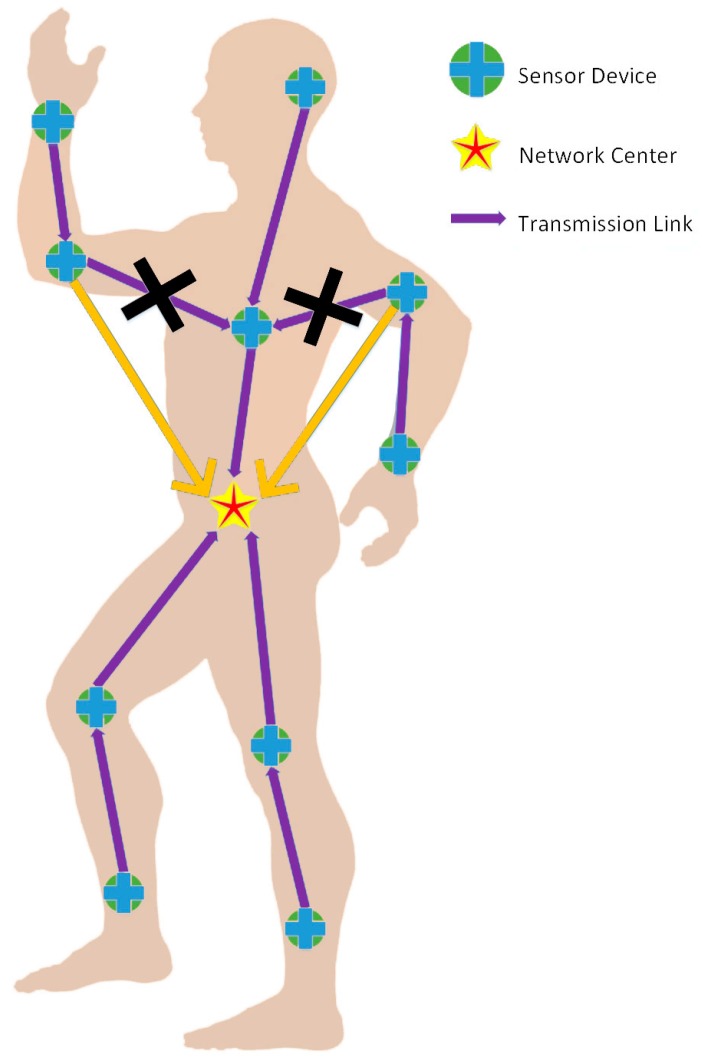
An instance of network topology optimization.

As Figure 6 shows, the sensor node just above the network center receives data packets transmitted from three other nodes. Other nodes, however, only receive packets from another one at most. Nevertheless, such imbalance can be mitigated through optimizing the network topology, for instance, let yellow links replace two original links covered by crosses. Hence, the optimization of network topology is required in order to achieve the equilibrium of consumed energy within a WBAN.

#### 4.1.4. Observation 4

**Figure 7:**
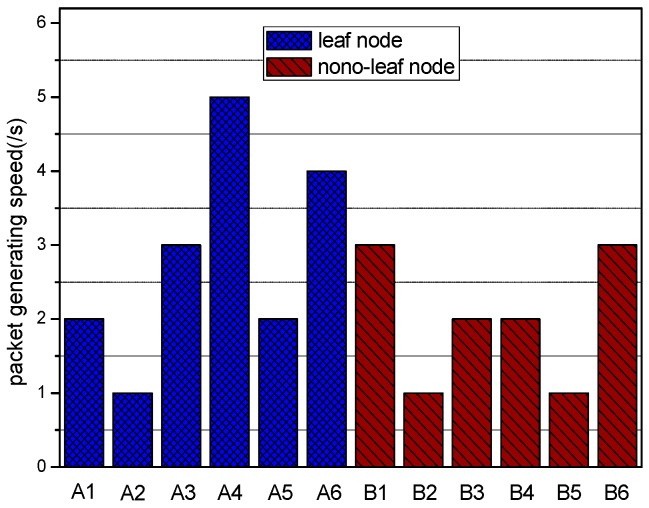
Packet generating speed of sensor nodes.

**Figure 8:**
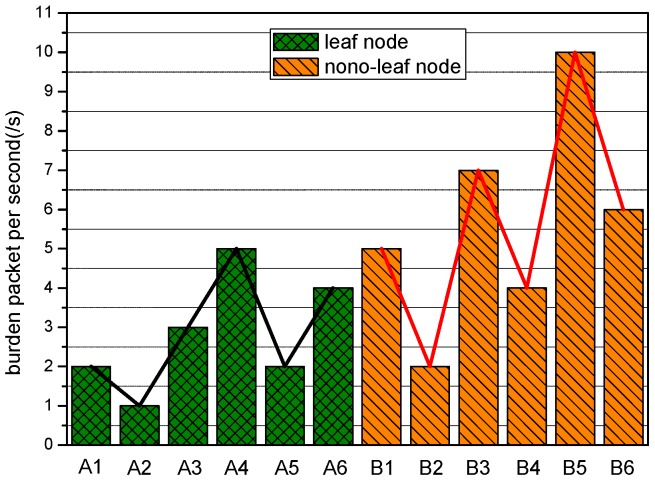
Packet burdens of sensor nodes.

Even though a scheme for relay decisions to balance the energy consumption distribution equilibrium exists, remaining energy still exists within the leaf nodes of the tree topology, since they are only responsible for the data sensed by themselves. Hence, if the remaining energy is utilized to enhance power further, delay and jitter will surely be reduced. Besides, unchanged network lifetime and fixed topology are guaranteed since further energy utilization is grounded on them at the very beginning. Consequently, power re-optimization for leaf nodes is expected to further improve the performance of WBAN after completing other optimizations. As shown in Figure 7 and Figure 8, though packet generating speed varies randomly no matter whether the transmitter is a leaf node or not, the packet burden of leaf nodes per second is quite less than that of none-leaf nodes on average.

Based on the observations above, our CLDO scheme is designed as the following way: first of all, transmission power over a single link can be chosen to maximize its energy efficiency. Then, a relay decision algorithm that takes energy efficiency and energy consumption balance into consideration is applied. After that, as to leaf nodes in the topology of the WBAN, their transmission power is further enhanced on the basis of the initial chosen power to fully utilize the remaining energy and raise the transmission reliability of the WBAN. Finally, an optimal packet size is selected to maximize the overall energy efficiency in the WBAN.

### 4.2. Optimal Power Control Strategy over Links

In this subsection, an optimal power control strategy is proposed for energy efficiency maximization. To begin with, energy efficiency is introduced to assess the quality of consumed power over a link. Then end-to-end delay and jitter are explored as the representatives of QoS requirements in the WBAN. After that, the power control strategy is described to maximize energy efficiency while QoS requirements are fulfilled. Finally, the theoretical value of the optimal power is obtained by formula derivations, along with a proposition to prove its existence.

According to our power control strategy, transmission reliability and energy consumption are two critical factors. Hence, the measurement on transmission reliability is introduced first. In a real-world WBAN, real-time SNR is typically unknown to devices because of the severe signal attenuation near the human body. As a result, measures computed according to a specific SNR cannot be a suitable criterion of transmission reliability, e.g., packet error rate, whereas, packet outage probability (POP) is a meaningful assessment of transmission reliability in realistic channels, due to the fact it signifies the fading realization with a guaranteed packet error rate $e^{p*}$. The POP of packets transmitted from node i to node j is [7]:

(5) $$POP_{i,j}=Pb\left(e_{i,j}^{p}>e_{i,j}^{p*}\right)=\omega \left(\frac{{\left[\frac{R_{b}}{W}\ln{\left(2-2\sqrt[{N}]{1-e_{p}^{*}}\right)}^{-1}\right]}^{\mathrm{dB}}-\mu_{i,j}}{\sigma_{i,j}}\right)$$

where Pb, ω, N, $R_{b}$, and W are the logogram of probability, the cumulative distribution function of the standard normal distribution, packet size, transmission bit rate, and channel bandwidth respectively. $e_{i,j}^{p}$ is packet error rate, computed as [18]:

(6) $$\;e_{i,j}^{p}=1-{\left[1-\frac{1}{2}\exp\left(\frac{-W}{R_{b}}\gamma_{i,j}\right)\right]}^{N}$$

Besides, the ratio between effective throughput and transmission power is widely used to measure energy efficiency for networks with limited energy [12,23]. Hence, provided that node’s relay is designated, consumed power $P_{i,j}$ over the link from node i to node j can be evaluated by an efficiency function, which is expressed as:

(7) $$\eta_{i,j}=\frac{R_{b}\left(1-POP_{i,j}\right)}{P_{i,j}}$$

The energy efficiency signifies the conservative number of bits successfully forwarded over the link(i,j) for per Joule of transmitter’s consumed energy with a guaranteed packet error rate. Hence, Equations (5) and (7) can be considered as basic assessments of power. Nevertheless, in order to optimize transmission power, some QoS requirement of the WBAN should be fulfilled, e.g., end-to-end delay constraint and jitter constraint. Therefore, before formally presenting the power control strategy, we first explore the end-to-end delay and jitter over links in the following paragraphs.

In a GI/G/1 queue, average end-to-end delay and jitter depend on the inter-arrival time and service time distribution. Inter-arrival time of a node combines the inter-arrival time of packets generated by itself and that of packets received from other nodes. We denote the inter-arrival distribution of packets at node n and that of self-generating packets at n as $AD_{i}$ and $AD_{i}^{self}$ respectively. Besides, as per our system model, $AD_{i}^{self}$ is exponentially distributed. Therefore, $E\left(AD_{i}^{self}\right)$ and $D\left(AD_{i}^{self}\right)$ are $v_{i}^{-1}$ and $v_{i}^{-2}$, respectively. Besides, based on the work done in [12], expected value $E\left(AD_{j}\right)$ and variance $D\left(AD_{j}\right)$ of $AD_{i}$ can be obtained as follows:

(8) $$E\left(AD_{j}\right)=E\left(AD_{j}^{self}\right)+\underset{r_{i}=j}{\sum }E\left(AD_{i}\right)\;$$

(9) $$D\left(AD_{j}\right)=D\left(AD_{j}^{self}\right)+\left[1-E\left(AD_{j}^{self}\right)\right]\underset{r_{i}=j}{\sum }E\left(AD_{i}\right)-{\left[\underset{j=r_{i}}{\sum }E\left(AD_{i}\right)\right]}^{2}+\underset{r_{i}=j}{\sum }\underset{\underset{and\;i^{\prime }\neq i}{r_{i^{\prime }}=j\;}}{\sum }E\left(AD_{i}\right)E\left(AD_{i^{\prime }}\right)\;\;+\underset{r_{i}=j}{\sum }\left\{D\left(SD_{i,j}\right)-E{\left(SD_{i,j}\right)}^{2}+2E\left(SD_{i,j}\right)E\left(AD_{i}\right)+\left[1-\delta_{i,j}\right]\left[D\left(AD_{i}\right)+E{\left(AD_{i}\right)}^{2}\right]\right\}\;$$

where $SD_{i,j}$ refers to the service time distribution of packets transmitted over link(i,j), and $\delta_{i,j}$ is a factor of utilized time for service, denoted as $\frac{E\left(SD_{i,j}\right)}{E\left(AD_{i}\right)}$.

Similarly, the expected value and variance of the service time over link(i,j) are given as follows [7]:

(10) $$E\left(SD_{i,j}\right)=\frac{8N}{3R_{b}}\left(\frac{2}{\tau_{i,j}}-3\tau_{i,j}+2\tau_{i,j}^{2}\right)$$

(11) $$D\left(SD_{i,j}\right)=\frac{N^{2}}{9R_{b}^{2}}\left(\frac{256}{\tau_{i,j}^{2}}-\frac{48}{\tau_{i,j}}+768-1976\tau_{i,j}+528\tau_{i,j}^{2}+768\tau_{i,j}^{3}-256\tau_{i,j}^{4}\right)$$

where $\tau_{i,j}$ denotes the conservative probability of successful transmission over link(i,j), with considerations of lost packets in signal collision case and false transmission case, and is computed by:

(12) $$\tau_{i,j}=1-\left[col_{i}+\left(1-col_{i}\right)e_{i,j}^{p*}\right]$$

$col_{i}$ is the collision probability of packets transmitted from node i, depicted in:

(13) $$\;col_{i}=1-\underset{i\in S\left\{i,hub\right\}}{\prod }\left(1-\delta_{i,j}\right)$$

Note that in Equation (13), collisions re considered in the whole topology, as each node is located in the carrier sensing range of any other node because of the WBAN’s small scale. Besides, $\delta_{i,j}$ is also the probability with which packets are transmitted over link(i,j).

Based on the inter-arrival time and service time obtained above, average end-to-end delay $L_{i,j}$ and jitter $J_{i,j}$ over link(i,j) can be approximated as below [32]:

(14) $$\;L_{i,j}=E\left(SD_{i,j}\right)+\frac{E\left(AD_{i}\right)D\left(SD_{i,j}\right)+E\left(SD_{i,j}\right)D\left(AD_{i}\right)}{2\left[1-E\left(AD_{i}\right)E\left(SD_{i,j}\right)\right]}$$

(15) $$\;J_{i,j}=\sqrt{\frac{E{\left(AD_{i}\right)}^{2}D\left(SD_{i,j}\right)+D\left(AD_{i}\right)E{\left(SD_{i,j}\right)}^{2}}{4E\left(AD_{i}\right)E\left(SD_{i,j}\right)}+\frac{D{\left(AD_{i}\right)}^{2}D\left(SD_{i,j}\right)+D\left(AD_{i}\right)D{\left(SD_{i,j}\right)}^{2}}{{\left[D\left(AD_{i}\right)+D\left(SD_{i,j}\right)\right]}^{2}}}$$

Furthermore, in a multi-hop path, the accumulated delay and jitter are calculated as follows:

(16) $$L_{i,hop}=\;L_{i,r_{i}}+L_{r_{i},hop}=\underset{j\in Route_{i}}{\sum }L_{j,r_{j}}$$

(17) $$J_{i,hop}=\;J_{i,r_{i}}+J_{r_{i},hop}\;=\underset{j\in Route_{i}}{\sum }J_{j,r_{j}}$$

where $r_{i}$ is the relay of node i, and $Route_{i}$ refers to the multi-hop path from node i to the hub.

Thus far, one assessment of transmission power and two measures of QoS has been obtained. Besides, in WBAN, the main target of power control, is to maximize the power while fulfilling the QoS requirements. Accordingly, for a given WBAN topology, the transmission power over link(i,j) can be decided according to the following optimization:

(18) $$\underset{P_{i,j}}{\max}\;\eta_{i,j}\;s.t.\;\{\begin{array}{c} L_{i,hub}\leq L_{i}^{*}\\ J_{i,hub}\leq J_{i}^{*} \end{array}$$

$L_{i}^{*}$ and $J_{i}^{*}$ are prescribed upper bounds of end-to-end delay and jitter in the multi-hop path from node i to the hub, which corresponds to the QoS requirements in the network. As the results in the equations above suggest, delay and jitter can be expressed as functions of the average SNR in the channel, which means that minimizing them is equivalent to maximizing the average SNR. Moreover, the specific relation between power and average SNR is:

(19) $${\bar{\gamma }}_{i,j}=\frac{G_{i,j}P_{i,j}}{N_{0}W}$$

where $G_{i,j}$ and $N_{0}$ refer to the channel gain over link(i,j) and thermal noise spectral density, respectively.

Hence, the optimization of transmission power can be expressed as below:

(20) $$\max_{{\bar{\gamma }}_{i,j}}\;\eta_{i,j}\;s.t.\;\{\begin{array}{c} {\bar{\gamma }}_{i,j}\geq \gamma_{i,j}^{*,L}\\ {\bar{\gamma }}_{i,j}\geq \gamma_{i,j}^{*,J} \end{array}$$

In order to obtain the solution to the optimization above, the maximization of $\eta_{i,j}$ should be explored with no restriction at first, whose expression accords to Equations (5)–(7) and (19) as a function of ${\bar{\gamma }}_{i,j}$:

(21) $$\max\;\frac{G_{i,j}R_{b}\left(1-\omega \left[Y\left({\bar{\gamma }}_{i,j}\right)\right]\right)}{{\bar{\gamma }}_{i,j}N_{0}W}$$

where:

(22) $$Y({\bar{\gamma }}_{i,j})=\frac{10\log_{10}\left[\frac{R_{b}}{W{\bar{\gamma }}_{i,j}}\ln{\left(2-2\sqrt[{N}]{1-e_{p}^{*}}\right)}^{-1}\right]}{\sigma_{i,j}}$$

Taking the derivative of the function above and equating it to zero, we derive that $\eta_{i,j}$ is maximized when ${\bar{\gamma }}_{i,j}$ equals to ${\overset{=}{\gamma }}_{i,j}$ constrained by:

(23) $$1-\omega \left[Y\left({\overset{=}{\gamma }}_{i,j}\right)\right]=\frac{10}{\sqrt{2\pi }\ln\left(10\right)\sigma_{i,j}}\exp\left[\frac{-Y{\left({\overset{=}{\gamma }}_{i,j}\right)}^{2}}{2}\right]$$

Note that in Equation (23), the left side is monotonically decreasing as ${\overset{=}{\gamma }}_{i,j}$ rises, while the right side is monotonically increasing at the same time. Besides, the ranges of the two sides overlap. Hence, as to Equation (23), there is a unique root ${\overset{=}{\gamma }}_{i,j}$ given to ${\bar{\gamma }}_{i,j}$ over link(i,j). When ${\bar{\gamma }}_{i,j}>{\overset{=}{\gamma }}_{i,j},$ the derivative of $\eta_{i,j}$ will not exceed zero, indicating that $\eta \left({\bar{\gamma }}_{i,j}\right)\leq \eta \left({\overset{=}{\gamma }}_{i,j}\right)$, which proves that further power enhancement has disadvantages to $\eta_{i,j}$.

Furthermore, ${\overset{=}{\gamma }}_{i,j}$ is derived in unconstrained conditions. However, as mentioned above, delay and jitter constraints with respect to the lower bound of ${\bar{\gamma }}_{i,j}$ have to be satisfied during the optimization of power control, leading to a possibility that ${\overset{=}{\gamma }}_{i,j}$ can be infeasible. As a result, power should be selected to maintain the average SNR ${\bar{\gamma }}_{i,j}={\dddot{\gamma }}_{i,j}$ for transmission over link(i,j) where:

(24) $${\dddot{\gamma }}_{i,j}=\max\left\{{\overset{=}{\gamma }}_{i,j},\;\gamma_{i,j}^{*,L},\gamma_{i,j}^{*,J}\right\}$$

Based on the description and analysis above, a formal proposition is provided to prove the existence of optimized power using aforementioned methods:

**Proposition** **1.** *If the condition* $e_{i,j}^{p*}<1-2^{-N}$ *is guaranteed for each link*(i,j) *in the WBAN, then the optimal power* $P_{i,j}^{opt}=\frac{{\dddot{\gamma }}_{i,j}N_{0}W}{G_{i,j}}$ *can be obtained.*

**Proof.**  When $e_{i,j}^{p*}<1-2^{-N}$, the definition of $Y({\bar{\gamma }}_{i,j})$ is meaningful. In this case, it is feasible for ${\bar{\gamma }}_{i,j}$ to be same as ${\dddot{\gamma }}_{i,j}$, so that the optimization of energy efficiency over each link(i,j) is achieved. Hence, according to Equation (19), the corresponding transmission power is $P_{i,j}^{opt}=\frac{{\dddot{\gamma }}_{i,j}N_{0}W}{G_{i,j}}$. □

### 4.3. Optimal Relay Decision Strategy for Sensor Nodes

In this subsection, an optimal relay decision strategy is proposed. In the beginning, the energy efficiency on route and energy consuming speed of a node are explored. Then, based on the efficiency and speed consumption, a candidate assessment function is introduced to select relays for sensor nodes. Afterwards, the detailed steps of the relay decision strategy are presented and a proposition is given to validate the effectiveness of the strategy. In the end, we summarize our strategy in the form of an algorithm.

In Section 4.2, the optimal power control strategy is adequately explored for an established transmission link(i,j), i.e., the strategy can only be implemented on sensor nodes that have already selected their relays. Therefore, the next step of the CLDO scheme is to determine the strategy for selecting relays for each sensor node, or to construct the topology of the WBAN.

When a sensor node chooses to transmit through a multi-hop path instead of direct transmission to the hub to avoid severe signal attenuation on the route, a relay or relays are required. The relays, however, lead to transmissions of the same packet in the WBAN. As a result, overall delay and jitter for transmission of a specific packet are expected to rise. Hence, a relay decision is vital for improving the performance of a WBAN. In order to resolve this problem, we consider enhancing the energy efficiency as much as possible while balancing the distribution of energy consumption through fully utilizing the remaining energy in nodes when the WBAN is declared dead.

In Equation (7), the evaluation function of energy efficiency has already been given, and it can be used here to help decide the relays, whereas due to the fact that the number of links in a multi-hop path is equivocal, the overall POP and total consumed power of the total path should be more appropriate than that of a single link when selecting a desired relay candidate.

As a result, a modified function that enables power efficiency to be evaluated in the path for relay decision is proposed, depicted as below:

(25) $$\eta_{i,j}^{rt}=R_{b}\times \frac{\prod_{k\in route_{i\left(j\right)}}\left(1-POP_{k,r_{k}}\right)}{\sum_{k\in route_{i\left(j\right)}}P_{k,r_{k}}}$$

where $\eta_{i,j}^{rt}$ refers to the overall power efficiency on the route $route_{i\left(j\right)}$ from sensor node i to relay node j, then to the hub, while $r^{k}$ is the direct relay of sensor node k.

Note that in Equation (25), though the overall transmission path is taken into consideration, it is utilized to select the optimal relay j of node i, i.e., for relay candidates of node i, their respective routes to the hub and the optimal powers consumed on the routes have been decided before calculating the power efficiency of node i, which mainly determines the value of $\eta_{i,j}^{rt}$. Based on the description above, such a scene could happen if $\eta_{i,j}^{rt}$ is taken as the only measure in our relay decision strategy.

Several sensor nodes, having not chosen their relays, share a candidate Cand with a relatively high overall power efficiency value; in turn, all of them prefer to transmit packets through Cand, leading to excessive packet accumulation in its transmitting queue. As a result, end-to-end delay and jitter are inevitably raised according to Equations (14) and (15). Besides, Cand has no choice but to increase its energy consumption to transmit packets due to the growth of the total data size. Other candidates, however, are ignored even if their queues are not crowded. Therefore, they consume energy at a limited rate. Hence, this kind of relay decision will produce an unbalanced distribution of energy consumption in the WBAN. Consequently, the data size burden of candidates should be involved in any relay decision strategy to achieve a more balanced energy consumption distribution.

Since the transmission rate is constant, when j is selected to be the relay of node i, why are nodes sometimes italic font and other times not—pick and use a consistent style the energy consumption speed of candidate j for transmitting burden data can be approximated by the product of the optimized power $P_{j,r_{j}}$ over link$\left(j,r_{j}\right)$ and time spent on transmitting total data that arrives at j per second, which includes data generated by itself, the received data from node i, and the collected data from other sensor nodes. Therefore, the energy consuming speed is depicted as:

(26) $$B_{i,j}=P_{j,r_{j}}^{opt}\times \frac{\left(Nv_{j}+Nv_{i}+\sum_{r_{k}=j}Nv_{k}\right)}{R_{b}}$$

Combining the evaluation function of power efficiency on the transmission route and data size burden by candidates, a candidate assessment function is derived to assess the fitness of candidate j to be the relay $r_{i}$ of sensor node i:

(27) $$Sel_{i,j}=\eta_{i,j}^{rt}+\frac{Z}{B_{i,j}}$$

where Z is the relative impact factor whose value reflects that the selection focus more on power efficiency or the equilibrium of consumed energy in WBANs.

The definition of $Sel_{i,j}$ indicates that high power efficiency on the route and low burden data size of the candidate are welcome in relay decision. Hence, the selection is equivalent to choosing the crest value of $Sel_{i,j}$:

(28) $$Sel_{i,r_{i}}=\max\left\{Sel_{i,j}\right\}\;\mathrm{where}\;j\in cad\left(i\right)$$

where cad(i) refers to the set of candidates in the relay decision of sensor node i. Due to the small scale of the WBAN, all sensor nodes except for itself have the potential to be its relay. However, there is no need for one sensor node to choose another node that is farther from the hub as its relay. Therefore, let d(i,j) denote the physical distance between device i and j, and cad(i) can also be expressed as $\left\{j_{i}\right\}$ where the distance constraint $d\left(j_{i},hub\right)<d\left(i,hub\right)$ is satisfied.

In the CLDO scheme, we aim to guarantee high energy efficiency and balanced consumed energy distribution in the WBAN. Hence, the specific procedure of topology formation can be described as follows:

(1) Initialize a star topology for the network, centered at the hub.
(2) Sort sensor nodes within the network into a queue according to the ascending order of their distance to the hub.
(3) Pick the head of the queue every time and implement a relay decision strategy for it until the queue is empty.
(4) Return to step (2) to start the next round of relay reselection.

The method of constructing topology described above is conducted in an iterative way. To make the method of selecting relay clearer, our relay decision (RD) scheme is described as Algorithm 1.

**Algorithm 1** Relay Decision (RD) Algorithm for Forming Topology1: input one value as the iteration time irt2: **For**
i∈S/{hub}3: $r_{i}=hub$; //initialize a star network topology4: cad(i)=S/{i,hub}; //obtain relay candidates5: **End**6: **While**
irt≠07: sort elements in S/{hub} into a queue Q in an ascending order of distance8: **While**
Q is not empty9: pull out the head i of Q;10: **For**
j∈cad(i)11: assume an established link(i,j) for transmission;12: compute the optimized power $P_{i,j}^{opt}$;13: compute the candidate assessment function $Sel_{i,j}$;14: **End**15: choose node $r_{i}$ as the relay of i that satisfies $Sel_{i,r_{i}}=\max\left\{Sel_{i,j}\right\}$ where j∈cad(i)16: **End**17: irt=irt−1;18: **End**

### 4.4. Power Rearrangement Strategy and Packet Size Choice Strategy

In Section 4.4, a power rearrangement strategy and packet size choice strategy are proposed. Besides, our CLDO scheme is proposed here. Firstly, the power rearrangement strategy is obtained to fully utilize the energy in sensor nodes and another proposition is given to show that QoS requirements are still fulfilled. After that, the impact of packet size on the performance of the WBAN is introduced. In the end, an algorithm is proposed to illustrate the CLDO scheme that achieves a comprehensive optimization of energy efficiency and energy balance in the WBAN.

In Section 4.2 and Section 4.3, a power control strategy based on a single transmission link and a relay decision strategy for determining the topology of the network have been proposed. Nevertheless, since the network lifetime mainly depends on the first dead node, there still remains much energy in sensor nodes when the network is declared dead, especially in those nodes that just transmit their own sensed data, i.e., the leaf nodes in the topology.

Hence, it is reasonable to further enhance the optimized power of leaf nodes, since fully utilizing the remaining energy enables the network to fulfill higher QoS requirements, such as delay, jitter, and transmission reliability. Besides, as leaf nodes will not act as relays of any other node, they are able to rearrange power while avoiding influencing other nodes.

In order to meet the requirement above, the energy consumption speed of leaf nodes should be rearranged as close as possible to the maximum consumption speed within the WBAN. We denote the energy consumption speed as $B_{i,j}^{re}$ when the rearranged power $P_{i,j}^{re}$ is adopted over the link(i,j). According to the energy consumption speed given in Equation (26), for one leaf node i, the power rearrangement strategy over link(i,j) can be simply expressed as the problem of resolving the following optimization problem:

(29) $$\min\left(B_{i,j}^{re}-\max B_{i,j}\right)\;s.t.\;B_{i,j}\leq B_{i,j}^{re}\leq \max B_{i,j},\;i\neq r_{k}\;\mathrm{where}\;k\in \frac{S}{\left\{hub\right\}}$$

Note that delay and jitter constraints are not mentioned in the power rearrangement strategy, in fact, they are implicit in the consumption speed constraint $B_{i,j}\leq B_{i,j}^{re}\leq \max B_{i,j}$, as $B_{i,j}\leq B_{i,j}^{re}$ indicates the enhancement of transmission power, which ensures less delay and jitter.

So far, a power control strategy, relay decision strategy and power rearrangement strategy have been introduced to optimize the performance of a WBAN. All the strategies above are based on the assumption that the packet size is given. As we know, sensor nodes tend to add a header to include essential information for smoothing the communication of each packet. Moreover, since transmissions are standardized by transport protocols employed in layers, the header size is fixed no matter how large the packet size is. As a result, the packet size determines the percent of sensed data size in a packet, whose variation has remarkable impacts on delay and jitter. Besides, packet size will influence the packet error rate in light of Equation (6), specifically, the packet error rate rises with the growth of packet size. Based on the description above, the optimal packet size can be obtained for improving the performance of a WBAN.

Due to the small scale of a WBAN, simultaneous transmission of any two nodes will cause communication collisions. Moreover, if their packet sizes are different, there will be difficulty in maintaining time synchronization of nodes in ALOHA. Hence, in order to ensure uniformity, our choice of packet size takes the overall WBAN into consideration. Assume that there is a set of packet sizes PS to choose from, the we denote the chosen size as $N_{c}$ in following parts.

To explore the specific impact of packet size on WBAN’s performance, firstly, we discuss its impact on the percent of sensed data in packets. For each packet, the context within it consists of two parts: the sensed data from body environment and header data, expressed as:

(30) $$N_{c}=D_{c}+H$$

where $D_{c}$ refers to the data size in a packet when the packet size is set to $N_{c}$, and H is the header’s data size.

Hence, in light of Equation (30), the percent of sensed data in a packet can be computed according to the following formula:

(31) $$percent\left(N_{c}\right)=\frac{D_{c}}{N_{c}}=\frac{N_{c}-H}{N_{c}}=1-\frac{H}{N_{c}}$$

Note that H is a constant. Therefore, the bigger the packet size is, the larger the percentage that the sensed data occupies. Hence, the method of computing the percentage has been obtained. It can be used in turn to derive the generation speed of sensed data. Since each node senses data at an individual speed that is irrelevant of packet size, the average packet generation speed of a node should be modified as per the sensed data consistency principle, that is:

(32) $$v_{i}\times Npercent\left(N\right)=v_{i}\left(N_{c}\right)\times N_{c}percent\left(N_{c}\right)$$

and converting it to the following form:

(33) $$\frac{v_{i}}{v_{i}\left(N_{c}\right)}=\frac{N_{c}-H}{N-H}$$

where $v_{i}\left(N_{c}\right)$ is the modified average packet generation speed when the packet size is set to $N_{c}$. The new performance of the WBAN with packet size variations can be derived through using $N_{c}$ and $v_{i}\left(N_{c}\right)$ to re-compute formulas based on the default packet size. Therefore, our packet size can be optimized to achieve the maximization of average energy efficiency $\eta_{mean}\left(N_{c}\right)$ on all transmission links in the WBAN. The optimization problem is expressed as:

(34) $$\underset{N_{c}}{\max}\;\eta_{mean}\left(N_{c}\right)$$

$\eta_{mean}\left(N_{c}\right)$ is calculated as:

(35) $$\eta_{mean}\left(N_{c}\right)=\underset{i\in S/\left\{hub\right\}}{\sum }\eta_{i,r_{i}}\left(N_{c}\right)/n$$

where $\eta_{i,r_{i}}\left(N_{c}\right)$ refers to the energy efficiency over link$\left(i,r_{i}\right)$ when the given packet size is $N_{c}$.

Based on the aforementioned description and the analysis related to power rearrangement strategy and packet size choice strategy, Algorithm 2 is proposed to illustrate a cross layer design optimization (CLDO) scheme, which is designed as below.

**Algorithm 2** Cross Layer Design Optimization (CLDO) Algorithm1:**For**
$N_{c}\in PS$2:employ RD scheme to form network topology and arrange consumed power;3:employ power rearrangement strategy to fully utilize remaining energy;4:compute average energy efficiency $\eta_{mean}\left(N_{c}\right)$;5:**End**6:choose the packet size that corresponds to the crest value of $\eta_{mean}\left(N_{c}\right)$ where $N_{c}\in PS$;7:implement the optimized power, the optimized topology obtained in step 1–4 that corresponds to the chosen packet size in WBAN.

So far, our CLDO scheme is presented to optimize energy efficiency and energy balance in WBANs based on the power control strategy, relay decision strategy, power rearrangement strategy, and packet size choice strategy.

## 5. Performance Analysis of CLDO Scheme

The theoretical performance of WBAN with CLDO scheme is analyzed in this section.

### 5.1. Transmission Reliability of CLDO Scheme

**Theorem** **1.** *In the CLDO scheme, the reliability of forwarding a packet from one node to the hub without any retransmission can be calculated as:*

(36) $$\partial =\underset{i\in route}{\prod }\left(1-\omega [Y({\bar{\gamma }}_{i,r_{i}},N_{C})]\right)$$

*where route refers to the multi-hop path starting from node* i *to the hub of WBAN, and the method for computing* $Y({\bar{\gamma }}_{i,r_{i}},N_{C})$ *is the same as for computing* $Y({\bar{\gamma }}_{i,r_{i}}),$ *except that* N *is replaced by* $N_{C}$ *.*

**Proof.**  Since the CLDO scheme chooses the optimal packet size $N_{C}$ rather than the default packet size N, $N_{C}$ should be included during the calculation of transmission reliability. The transmission reliability of a packet without any retransmission can be interpreted as the total successful transmission over each link on the route, i.e., packet outage never appears. Hence, according to Equations (5), (19), (21) and (22), the packet outage probability over link$\left(i,r_{i}\right)$ is expressed as $\omega \left[Y({\bar{\gamma }}_{i,r_{i}},N_{C})\right]$. $\left(1-\omega \left[Y({\bar{\gamma }}_{i,r_{i}},N_{C}\right)]\right)$ is the probability of forwarding a packet without retransmission over the link. Thus, the transmission reliability of a packet on the route is the accumulated probability of forwarding a packet over each link on the route, computed as the product of $\left(1-\omega \left[Y({\bar{\gamma }}_{i,r_{i}},N_{C}\right)]\right)$, which is shown in Equation (36). □

### 5.2. Energy Efficiency of CLDO Scheme

**Theorem** **2.** *In the CLDO scheme, the average energy efficiency over all the links within a WBAN is expressed as:*

(37) $$\varepsilon =\frac{R_{b}}{n}\underset{i\in S/\left\{hub\right\}}{\sum }\frac{\left(1-\omega \left[Y({\bar{\gamma }}_{i,r_{i}},N_{C}\right)\right])}{P_{i,r_{i}}^{opt}\left(N_{C}\right)}$$

*where* $P_{i,r_{i}}^{opt}\left(N_{C}\right)$ *is the optimized power in the power control strategy when the designated packet size is* $N_{C}$ *.*

**Proof.**  The energy efficiency over a single link$\left(i,r_{i}\right)$ is defined as the ratio of the transmitter’s throughput to the transmission power. As per Equations (7), (21) and (22), the efficiency can be depicted as $\frac{R_{b}\left(1-\omega \left[Y({\bar{\gamma }}_{i,r_{i}},N_{C}\right)\right])}{P_{i,r_{i}}^{opt}\left(N_{C}\right)}$. Besides, the topology of the WBAN constructed by the CLDO scheme is a tree topology, i.e., each sensor node launches one and only one link within the network, hence, the number of links equals the number of sensor nodes n. According to its definition, average energy efficiency should be computed via dividing the sum of energy efficiency over each link by the number of links, i.e., the right side in Equation (37). □

### 5.3. Energy Consumption Balance of CLDO Scheme

**Theorem** **3.** *In the CLDO scheme, the balance degree of consumed energy distribution is reflected by the ratio of the maximum value to the minimum value among nodes’ energy consumption speeds, depicted as below*:

(38) $$\theta =\frac{\min\left(P_{i,r_{i}}^{opt}\left(N_{C}\right)\times N_{C}\left[v_{i}\left(N_{C}\right)+\sum_{r_{k}=i}v_{k}\left(N_{C}\right)\right]\right)}{\max\left(P_{i,r_{i}}^{opt}\left(N_{C}\right)\times N_{C}\left[v_{i}\left(N_{C}\right)+\sum_{r_{k}=i}v_{k}\left(N_{C}\right)\right]\right)}\;\mathrm{where}\;i\in S/\left\{hub\right\}$$

**Proof.**  According to Equation (26), the energy consumption speed of a certain node can be approximated by the product of the transmission power and data size burden by the node per second. Furthermore, a node’s burden data size is composed of the self-produced data size and the size of the data sensed by previous nodes. Besides, the burden data size per second can be calculated as $N_{C}v_{i}\left(N_{C}\right)$, the result of multiplying packet size $N_{C}$ and the modified packet generation speed $v_{i}\left(N_{C}\right)$. Hence, the energy consumption speed is expressed as $P_{i,r_{i}}^{opt}\left(N_{C}\right)\times N_{C}\left[v_{i}\left(N_{C}\right)+\underset{r_{k}=i}{\sum }v_{k}\left(N_{C}\right)\right]$. Finally, the degree of consumed energy distribution balance is obtained using Equation (38). □

### 5.4. End-to-End Delay and Jitter of CLDO Scheme

**Theorem** **4.** *End-to-end delay and jitter of each node on their overall routes to the hub are smaller than their prescribed upper bounds in the CLDO scheme:*

(39) $$\{\begin{array}{c} L_{i,hub}\left(N_{C}\right)\leq L_{i}^{*}\\ J_{i,hub}\left(N_{C}\right)\leq J_{i}^{*} \end{array}$$

where $L_{i,hub}\left(N_{C}\right)$ and $J_{i,hub}\left(N_{C}\right)$ are the end-to-end delay and the jitter on the route from node i to the hub respectively when packet size is set to $N_{C}$.

**Proof.**  In the initial power control strategy, constraints $L_{i,hub}\leq L_{i}^{*}$ and $J_{i,hub}\leq J_{i}^{*}$ are satisfied for each sensor node in the WBAN. Therefore, in the RD scheme with respect to power control and topology formation, no matter how the network topology is shaped, the above condition is met. Moreover, in the CLDO scheme, the consumed powers of certain sensor nodes are further enhanced with a chosen packet size $N_{C}$, which lead to less end-to-end delays and jitters in the WBAN under the premise that two unequal relations still exist. Hence, constraints on delay and jitter are kept in the CLDO scheme, as Equation (39) shows. □

### 5.5. Network Lifetime of the CLDO Scheme

**Theorem** **5.** *In the CLDO scheme, if each sensor node is equipped with the same initial battery energy* $e_{battery}$*, then the lifetime of the WBAN is calculated as (in seconds):*

(40) $$\beta =\frac{e_{battery}}{\max\left(P_{i,r_{i}}^{opt}\left(N_{C}\right)\times N_{C}\left[v_{i}\left(N_{C}\right)+\sum_{r_{k}=i}v_{k}\left(N_{C}\right)\right]\right)}$$

**Proof.**  The network is declared dead when the first node with exhausted energy appears. Hence, the lifetime of the network can be interpreted as the consumption time β for the first dead node to run out of energy, which corresponds to the maximum energy consumption speed. Besides, the energy consumption speed of a sensor node is given in the proof of Theorem 3, i.e., $P_{i,r_{i}}^{opt}\left(N_{C}\right)\times N_{C}\left[v_{i}\left(N_{C}\right)+\underset{r_{k}=i}{\sum }v_{k}\left(N_{C}\right)\right]$. Therefore, consumption time β can be obtained through dividing the initial battery energy $e_{battery}$ by the consumption speed. Therefore, the lifetime of the WBAN can be proved as shown in Equation (40). □

## 6. Experimental Results and Analysis of the CLDO Scheme

In this section, experimental results are presented to compare the CLDO scheme with the relay selection and power control game (RSPCG) [7] and the direct transmission (DT) scheme. In the beginning, parameters settings for the WBAN system are introduced. After that, the experimental results are discussed according to these settings.

### 6.1. Experimental Parameter Settings

Considering the realistic physical environment of the human body, sensor nodes in WBANs are distributed according to Figure 1. Their pairwise distances and channel conditions are given in Tables 2 and 3 from [18], respectively. Available packet size ranges from 100 bits to 1000 bits, which takes 100 bits as an interval, and the power can be chosen in the [−60 dBm, −40 dBm] interval. The power attenuation model within dynamic channels is the same as in [18], described as follows:

(41) $$Atte_{i,j}=Atte_{\left(0\right)}+10k\times \log\left(\frac{d_{i,j}}{d_{0}}\right)+R$$

where $Atte_{\left(0\right)}$ is the power attenuation at the reference distance $d_{0}$, k is the path-loss exponent, and R is a random number used to reflect the impact of other factors within channels, ranging from 0 dB to 10 dB. $Atte_{\left(0\right)}$, k and $d_{0}$ are designated as 16.6 dB, 3.9 and 10 cm, respectively. Other parameter settings are presented in Table 1 with reference of the IEEE 802.15.6 impulse radio UWB (IR-UWB) PHY [7,51,52,53].

### 6.2. Experimental Results of the CLDO Scheme

**Figure 9:**
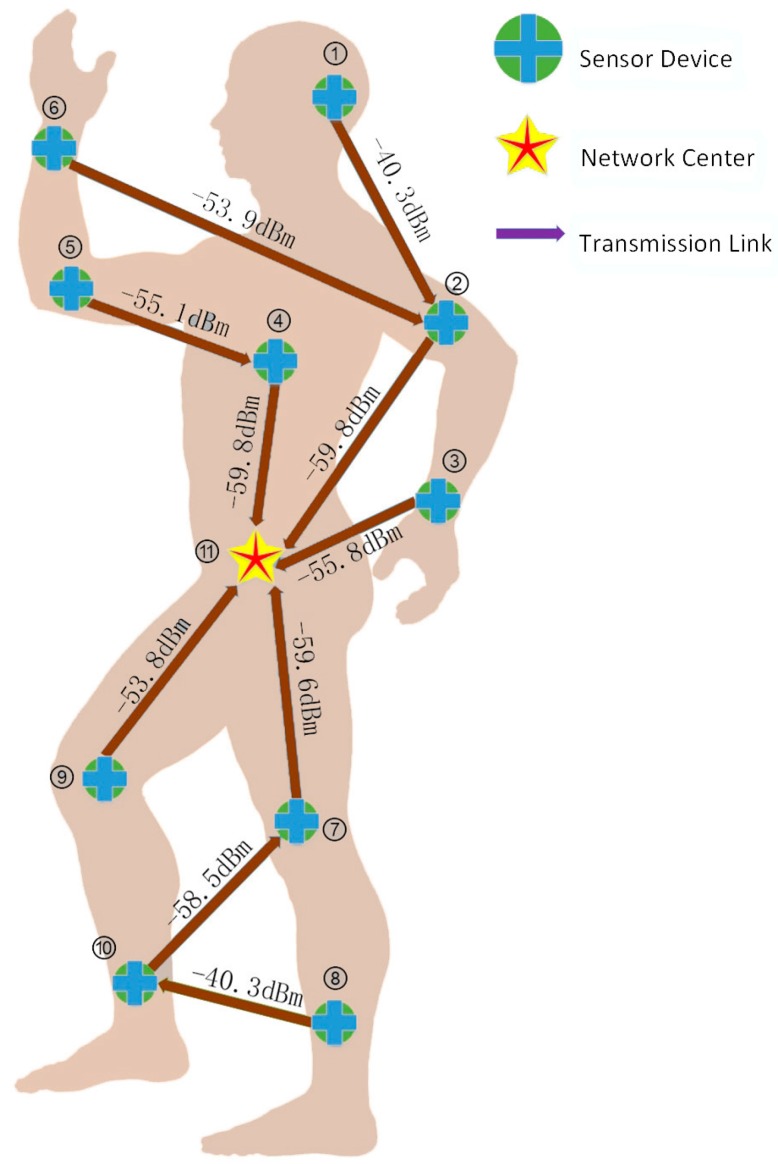
Experimental results of the CLDO scheme (chosen packet size is 200 bits).

Many of the experimental parameters (e.g., channel bandwidth and thermal noise spectral density) are set according to the IEEE 802.15.6 impulse radio UWB (IR-UWB) PHY. Figure 9 shows the power and relay decision results of our CLDO scheme implementation, and detailed information on the deployment of each sensor node is shown in Table 2.

As Figure 9 presents, due to the sharp signal attenuation in a relatively long path, sensor nodes prefer to select a relay and commit data packet to it for reliable transmission. The involvement of relay results in more successful transmission times for a certain packet. Hence, not only energy efficiency is enhanced with raised transmission reliability over links, but also low delay can be guaranteed due to the reduced number of transmission failures. Furthermore, we note that the power utilized for connecting links to the hub is lower than the power for links far from the hub, which violates the exact distribution of data load. This phenomenon corresponds to our balanced strategy for energy consumption speed in the CLDO scheme. Besides, the derived optimal packet size is only 200 bits, leading to unnegligible positive impacts of packet header on the total data flow in the WBAN, the end-to-end delay, and jitter. The reason for this packet size choice is that sensor nodes struggle to avoid packet loss under the severe fading environment on the human body.

In Table 3, different relay choice results of the CLDO scheme, RSPCG, and DT scheme are shown. As Table 3 presents, the CLDO scheme exploits more relay nodes to improve the energy efficiency compared with the DT scheme, where all sensor nodes are connected to the center directly. In addition, given the fact that RSPCG forges LeftArm nodes to transmit the data load of three nodes, Chest, LeftLeg and LeftFoot, while a node in the CLDO scheme is responsible for two nodes at most. Spontaneously, the conclusion can be drawn that our CLDO scheme forms a more energy-balanced topology for the WBAN than the RSPCG.

### 6.3. End-to-End Delay and Jitter of the CLDO Scheme

Table 4 shows the different end-to-end delay and jitter over single links and in multi-hop paths, respectively. For convenience, the links and paths are identified by their origin node. The so-called end-to-end delay refers to the theoretical delay under the condition that a packet error rate is prescribed, i.e., the selected power for sensor nodes plays no role here, as does jitter.

As Table 4 shows, the ideal end-to-end delay over a single link in the CLDO scheme exceeds that in both the RSPCG and DT schemes on average. A similar situation occurs with the comparison of the delay in multi-hop paths. This can be interpreted by the difference of extra data load in schemes. If delay constraints can be satisfied, our CLDO scheme tends to select light packets as a unit in data transmission to enhance transmission reliability on the route. Spontaneously, the total data load flowing in the WBAN will be raised since the header occupies a larger percentage with smaller packet size, which eventually leads to a growth of delay. Though the delays raised in the CLDO scheme, they are still smaller than 1 s, our delay threshold, i.e., QoS with respect to delay can be guaranteed. Thus, the delay variation in the CLDO scheme is acceptable. As to the analysis on jitter, presented jitters have the same pattern as the delay. Therefore, more detailed explanations about the jitter are unnecessary here.

### 6.4. Transmission Reliablity of the CLDO Scheme

**Figure 10:**
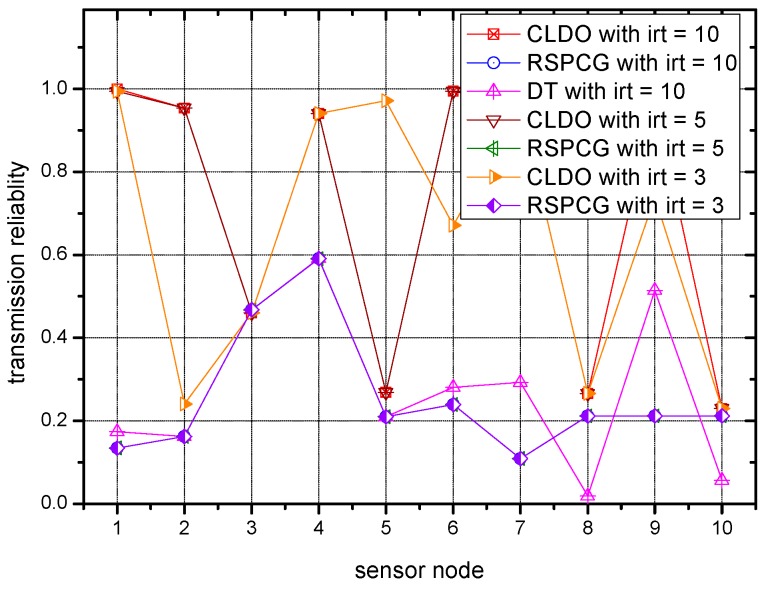
Transmission reliability over a single link with different numbers of iterations of the different schemes.

**Figure 11:**
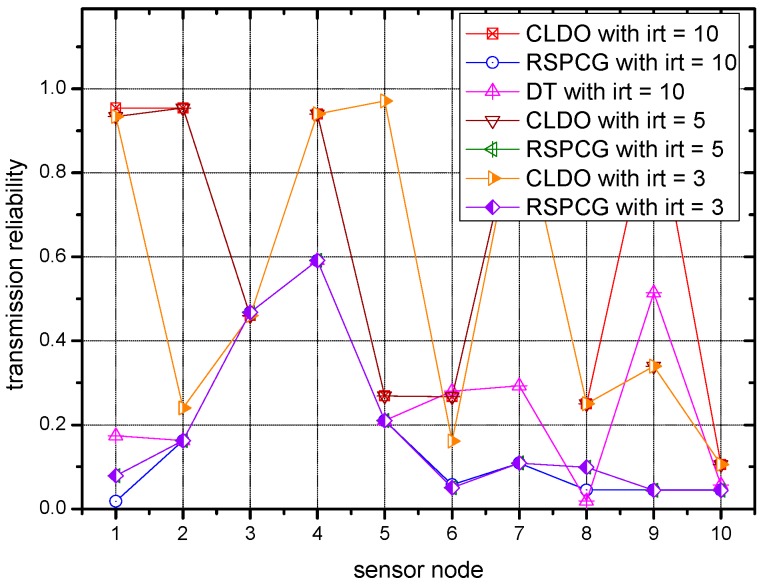
Transmission reliability in a multi-hop path with different numbers of iterations for different schemes.

Figure 10 and Figure 11 present the transmission reliability during packet transmission with different iteration times irt. Note that the numbers on *X*-axis correspond to the number of nodes in Table 2. As Figure 10 reflects, the transmission reliability of CLDO scheme over a single link is significantly higher (by 86.5% and 82.6%) than the reliability of the RSPCG and DT schemes when the number of iterations is 10. Such a huge promotion can mainly be attributed to the packet size selection in the CLDO scheme. As said before, due to the severe fading conditions on the human body, power control cannot satisfy the high requirements in WBANs, which is the core function of the RSPCG and DT schemes, whereas, aiming to maximize energy efficiency, our CLDO scheme has the capacity to adaptively decide a suitable packet size, a vital factor of transmission reliability. The specific degree by which the CLDO scheme exceeds to RSPCG in reliability is 44.6% and 85.9% on the average when the number of iterations is 5 and 3, separately. Besides, we note that for a certain scheme (except for the DT scheme), transmission reliability grows with the ascending number of iterations, verifying that with larger iteration numbers, the reliability performance can be further improved.

### 6.5. Energy Efficiency of the CLDO Scheme

**Figure 12:**
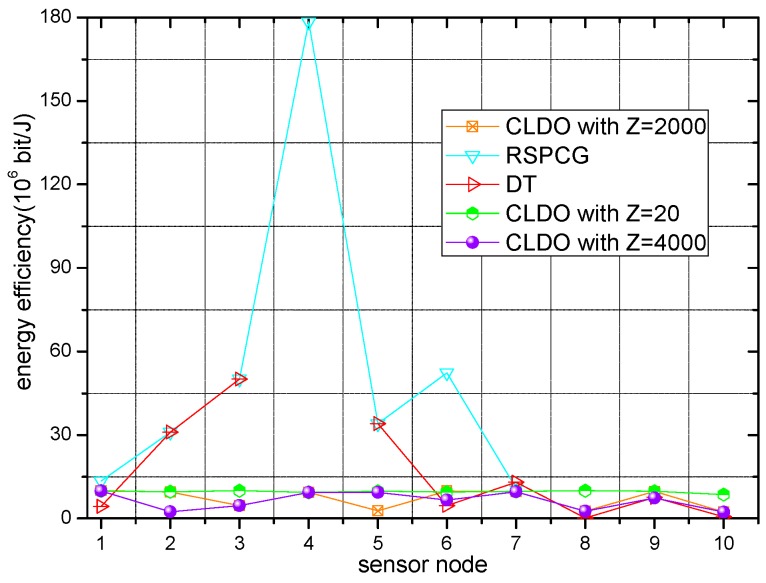
Energy efficiency over a single link.

**Figure 13:**
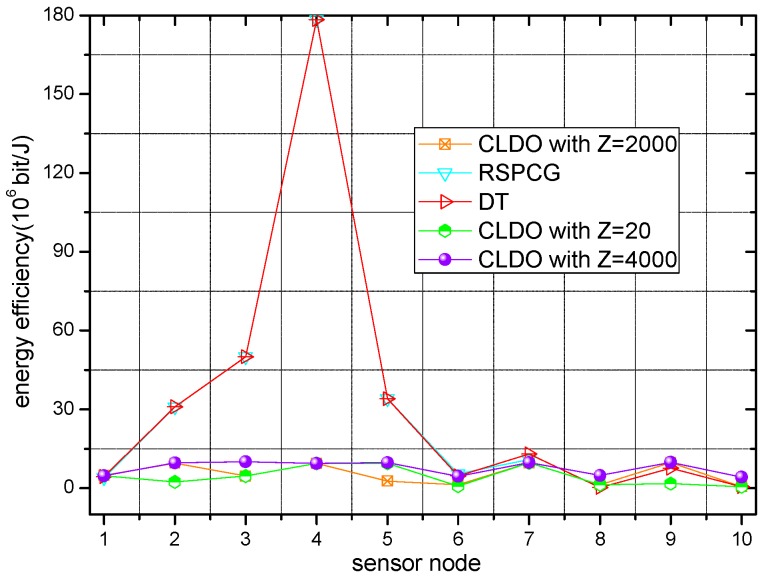
Energy efficiency in a multi-hop path.

Energy efficiency of different schemes with various relative impact factors Z over a single link and in a multi-hop path are presented in Figure 12 and Figure 13, respectively. As Figure 12 and Figure 13 show, the sensor nodes’ energy efficiency is a little higher in the RSPCG and DT schemes compared with the CLDO scheme since our CLDO scheme takes energy consumption balance into consideration in addition to energy efficiency. Note that we depict three curves for the CLDO scheme with various relative impact factors (20, 2000, 4000) in the two figures. Obviously, with the growth of relative impact factor, the overall energy efficiency of sensor nodes within the WBAN tends to decline, i.e., the importance of energy efficiency descends in the relay decision strategy. Furthermore, an overt discrepancy appears between the efficiency over a single link and in a multi-hop path, indicating that the overall efficiency of a sensor node is affected to a great degree by the efficiency over links within the path, but indirectly connected to it.

### 6.6. Energy Balance Degree of the CLDO Scheme

**Figure 14:**
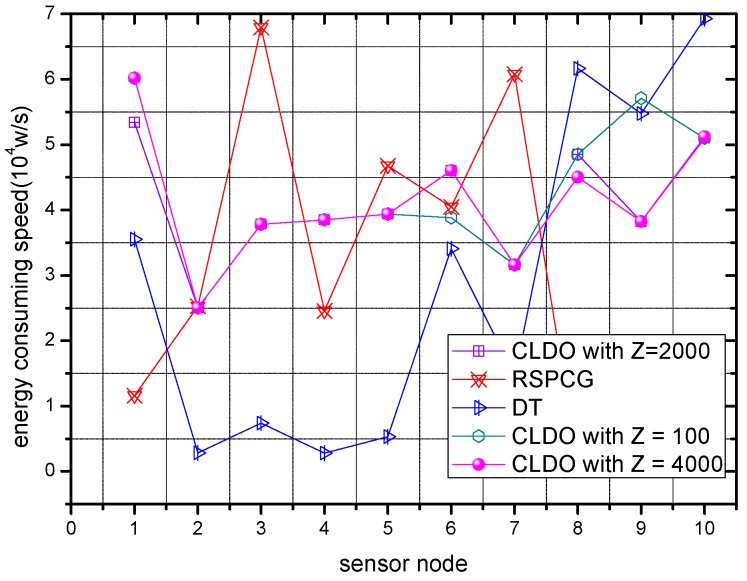
Energy consumption speed of sensor node in different schemes.

**Figure 15:**
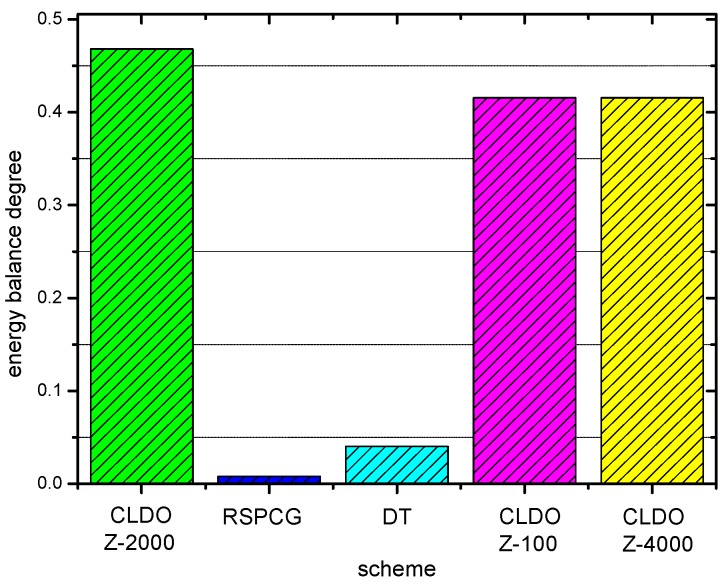
Energy balance degree of the WBAN in different schemes.

Figure 14 shows the energy consumption speed of sensor nodes within a WBAN in the three schemes. Obviously, the speed in the RSPCG and CLDO schemes is much higher than that in the DT scheme. Based on the aforementioned analysis of transmission reliability of the schemes in Section 6.4 and of energy efficiency in Section 6.5, we can infer that this phenomenon results from the incentive of nodes to choose the lowest power to cater to low transmission reliability for high energy efficiency in the DT scheme. In this way, though a WBAN can implement the DT scheme to maintain a working state for a long time with a slow energy consumption speed, effective packet transmission is limited during its entire life. Furthermore, it’s handy to discover that the energy consumption speed is distributed in a more balanced way in the CLDO scheme, which is consistent with our descriptions in Section 4 where the CLDO scheme was introduced. A more explicit comparison is presented in Figure 15, where the degree of energy balance of RSPCG is quite lower than that of the CLDO scheme, and the series of symbols Z-k stands for the value k provided a relative impact factor Z. Moreover, the degree of energy balance can also represent the minimum energy utilization rate of sensor nodes in the WBAN when it is declared to have died.

### 6.7. Lifetime of the CLDO Scheme

**Figure 16:**
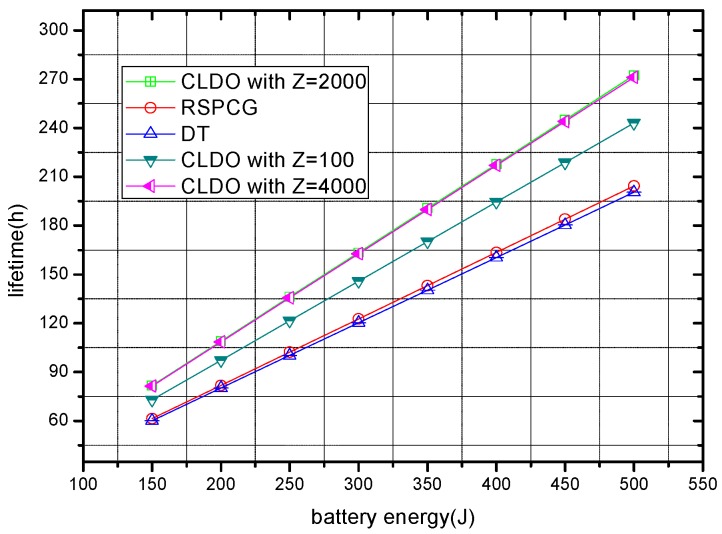
Lifetime of the WBAN with different battery energy-equipped sensor nodes.

Figure 16 shows the relation between battery energy of sensor nodes and lifetime of the WBAN in the different schemes. Saliently, the CLDO scheme provides a longer lifetime compared with the RSPCG and DT schemes (by 33.2% and 35.8%, respectively). This can be illustrated by the particular capacity of the CLDO scheme to balance the energy distribution to reduce the maximum energy consumption speed in the WBAN, and thus eventually prolong the network lifetime. Furthermore, we note that the CLDO scheme with a bigger relative impact factor Z has a longer network lifetime than the schemes with smaller Z, i.e., the growth of Z makes our CLDO scheme focus more on energy balance distribution, leading to reduced maximum energy consumption speed and a prolonged lifetime.

**Figure 17:**
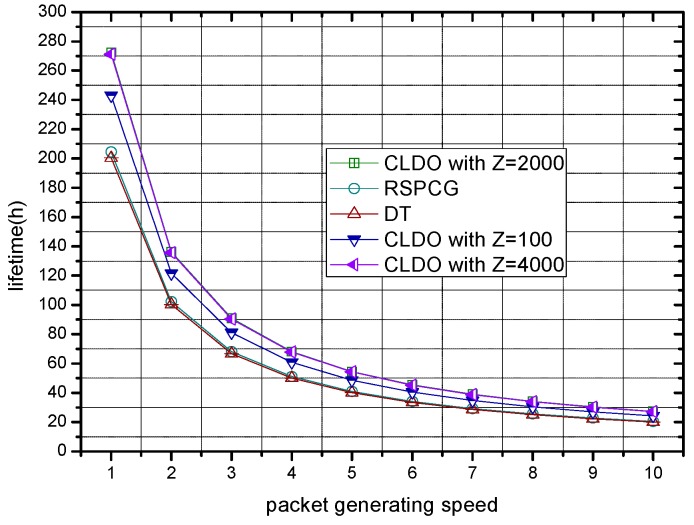
Lifetime of the WBAN with different packet generation speeds.

In Figure 17, the lifetime of WBANs with different packet generation speeds in the different schemes is provided (battery energy is set to 500 J). The so-called packet generation speed refers to the value given to the origin packet generation speed v. As Figure 17 shows, the lifetime is reduced with rising packet generation speed since the maximum energy consumption speed of sensor nodes is raised at the same time. Besides, it can be observed that our CLDO scheme still has a longer lifetime compared with the RSPCG and DT schemes when the packet generation speed varies.

### 6.8. Study on Body Movement

**Figure 18:**
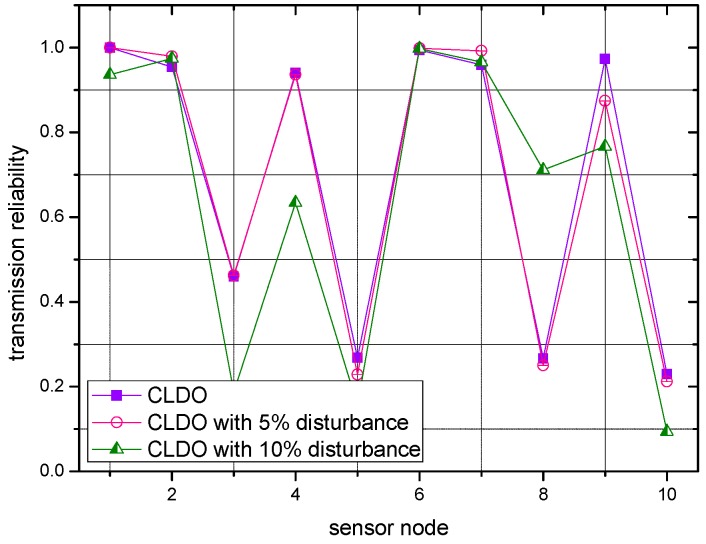
Transmission reliability over a single link with different disturbances.

**Figure 19:**
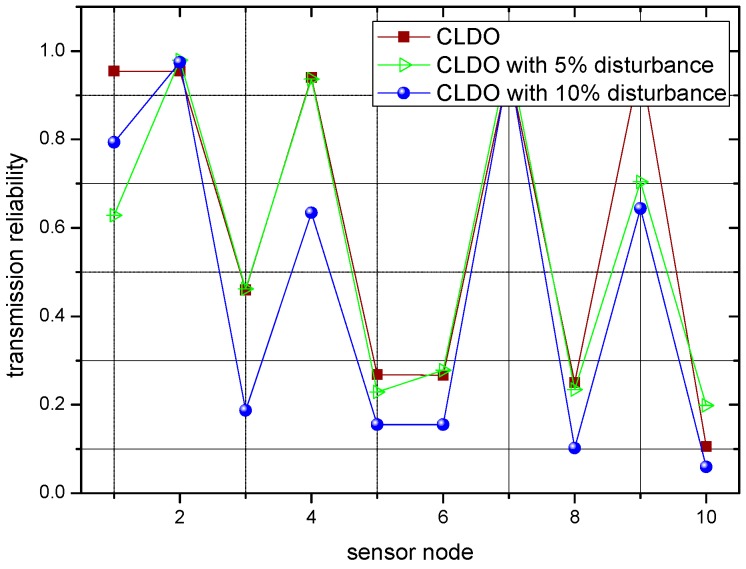
Transmission reliability over multiple links with different disturbances.

In previous experiments, the locations of sensor nodes were fixed. Since simulating body movements can be relatively complex, we just make slight changes to study the impact of body movements in following descriptions, namely by varying the value of the distance between any two sensor nodes in a random way and to a tiny degree, namely applying a disturbance. Figure 18 and Figure 19 present the transmission reliability of the CLDO scheme with different disturbances over a single link and over multiple links, respectively. 

As the figures show, though the disturbances cause a slight random variation in the transmission reliability, as long as it is not salient, e.g., 5% or 10%, the condition of transmission reliability within the WBAN remains nearly the same. Hence, our former experiment results make sense and our proposed CLDO scheme provides a comprehensive way to configure a WBAN to optimize transmission reliability, energy efficiency, and lifetime, it inevitably brings a slight overhead, which can be summarized as follows:

① CLDO has complex procedures during network initialization, which is costly in both consumption and time.
② The value of the number of iterations in the CLDO algorithm influences its performance to a considerable degree, but we cannot guarantee the rationality of its setting sometimes.
③ Since the network will be reinitialized whenever a sensor node enters or leaves the WBAN, therefore, the CLDO scheme lacks the capacity to deal with dynamic location situations, but from the overall view, the estimable contribution of the CLDO scheme totally warrants such a slight overhead.

## 7. Conclusions and Future Work

In this paper, we have proposed a novel cross layer design optimization (CLDO) scheme to enhance transmission reliability, improve energy efficiency, and prolong network lifetime in body sensor networks, grounded on power control, relay decision and packet selection within the WBAN. Different from previous studies on WBANs, our scheme adopts a cross layer design that involves the physical layer, MAC layer, and network layer. In our design, we firstly choose the optimal power consumption by maximizing the energy efficiency over a single link, and then decide the optimal relay through a trade-off between the maximization of energy efficiency and the minimization of energy consumption speed. After that, the remaining energy in leaf nodes of the WBAN topology is further utilized to enhance the transmission reliability without any loss of lifetime. Finally, the optimal packet size is selected for optimizing the energy efficiency. In the end, a simulation experiment is conducted, which shows that our CLDO scheme obtains better transmission reliability and lifetime performance compared with a relay selection and power control game (RSPCG) approach.

## Figures and Tables

**Table 1:** Parameter settings of the WBAN.

Symbol	Description	Value
N	Default Packet Size	800 bit
H	Packet Header Size	50 bytes
W	Channel Bandwidth	499.2 MHz
$e_{battery}$	Battery Energy	500 J
$N_{0}$	Thermal Noise Spectral Density	$4.057\times 10^{-21}$
g	Channel Gain	0.1
$R_{b}$	Transmission Bit Rate	487.5 kbps
Z	Relative Impact Factor	2000
$v_{i}$	Packet Generating Speed of Node i	3~5
$e_{p}^{*}$	Prescribe Packet Error Rate	0.01
$L^{*}$	Latency Threshold	1 s
$J^{*}$	Jitter Threshold	1
irt	Iteration Times	10

**Table 2:** Series Number of Sensor Nodes.

**Number**	(1)	(2)	(3)	(4)	(5)	(6)	(7)	(8)	(9)	(10)	(11)
**Node**	Head	LeftArm	LeftHand	Chest	RightArm	RightHand	LeftLeg	LeftFoot	RightLeg	RightFoot	Center

**Table 3:** Relay Choice of Sensor Nodes in Different Schemes.

	Head	LeftArm	LeftHand	Chest	RightArm	RightHand	LeftLeg	LeftFoot	RightLeg	RightFoot
CLDO	LeftArm	Center	Center	Center	Chest	LeftArm	Center	RightLeg	LeftLeg	LeftLeg
RSPCG	Center	Center	Chest	LeftArm	Center	Chest	LeftArm	LeftArm	RightArm	RightARm
DT	Center	Center	Center	Center	Center	Center	Center	Center	Center	Center

**Table 4:** End-to-End Delay and Jitter over Single Links and Multi-Hop Paths ($L_{i}\left[\mathrm{ms}\right]/J_{i}\left[\mathrm{ms}\right]$).

	Single Link			Multi-Hop Path		
	CLDO	RSPCG	DT	CLDO	RSPCG	DT
Head	7.30/33.29	5.80/37.58	6.92/28.79	171.20/137.46	12.01/63.9	6.92/28.79
LeftArm	163.89/104.18	6.10/32.73	7.25/35.97	163.89/104.18	6.13/32.73	7.25/35.97
LeftHand	417.59/137.74	6.39/38.3	6.99/30.06	417.59/137.74	6.44/38.3	6.99/30.06
Chest	607.40/161.5	6.10/38.49	6.96/29.31	607.40/161.5	6.19/38.49	6.96/29.31
RightArm	927.69/193.39	6.69/46.65	6.96/29.46	927.69/193.39	6.71/46.65	6.96/29.46
RightHand	16.79/41.49	6/31.88	7.25/32.35	944.50/234.88	12.79/78.53	7.25/32.35
LeftLeg	11.89/37.89	5.89/29.05	7.08/32.35	11.89/37.89	5.97/29.05	7.08/32.35
LeftFoot	13.79/39.37	6/30.21	7.13/33.36	621.19/200.87	12.45/68.51	7.13/33.36
RightLeg	8.29/34.44	5.80/26.32	6.99/30	8.29/34.44	12.57/72.97	6.99/30
RightFoot	11.50/37.58	5.89/28.8	7.07/31.73	429.09/175.32	12.67/75.45	7.07/31.73
